# Réforme et performance de l’Inspection de la santé et de la Division de la santé au Sud-Kivu en République Démocratique du Congo

**DOI:** 10.4102/phcfm.v12i1.2534

**Published:** 2020-12-15

**Authors:** Jean Louis Mopene, Christian Molima, Jean Bosco M. Kahindo, Samuel Makali, Hermès Karemere

**Affiliations:** 1 École Régionale de Santé Publique (ERSP), Faculté de Médecine, Université Catholique de Bukavu (UCB), Bukavu, Democratic Republic of Congo, Congo; 2 Faculté de Médecine, Université Officielle du Ruwenzori, Butembo; ULB Coopération, Goma, Democratic Republic of Congo, Congo; 3 Médecine Interne, Faculté de Médecine, Université Catholique de Bukavu (UCB), Bukavu, Democratic Republic of Congo, Congo

**Keywords:** Reform, Decentralization, Health system, Performance, South Kivu, Democratic Republic of Congo

## Abstract

Reform and Performance of the Provincial Health Inspectorate and the Provincial Division of Health of South Kivu in the Democratic Republic of Congo.

**Background:**

The intermediate level incorporated both the Provincial Health Inspectorate (IPS) and the Provincial Health Division (DPS) of Health. The new constitution of 2006 gave impetus to decentralisation, which became effective in 2015. The reform introduced at the intermediate level clearly separated the IPS and the DPS. This article assesses the effect of this reform on the performance of IPS and DPS in South Kivu, Democratic Republic of Congo.

**Methodology:**

The study is evaluative before and after and covers the period from 2012 to 2017. It uses mixed methods: three techniques were used to collect data including observation, document review and individual interviews. The analysis of the quantitative data concerned the evolution of the indicators; that of qualitative data was carried out by themes from two theoretical models: the ministerial functional framework and the ‘Strengths, Weaknesses, Opportunities and Threats’ analysis framework (SWOT analysis). Scores were assigned to each managerial function according to their level of performance for better comparison.

**Results:**

After the reform, a decline in the performance score of activities devolved to IPS is noted, mainly due to the low funding of activities. On the other hand, in the DPS, the evolution of the score is favorable, because of the strong support given to the reform at this level by the partners and the government. The alignment of partners to a single contract for funding DPS activities is observed. The weak financing of the health sector by the government remains a weak point, however, and the brain drain a threat to institutional sustainability. The introduction of the single financing contract constitutes an opportunity to improve the performance of the provincial management team.

**Discussion and Conclusion:**

The study shows the improvement in the performance of managerial functions of the DPS and the regression to the IPS. The low funding of IPS by the Congolese government could jeopardise the reform.

## Introduction

Depuis son accession à l’indépendance le 30 juin 1960, la RD Congo a traversé plusieurs périodes de crise. La plus récente perdure depuis trois décennies, caractérisée par des rebellions et des guerres récurrentes. Des dialogues ont été tenus entre le gouvernement de la RDC et des belligérants^1,2^ dont celui de Sun City en 2002^1,3^. Ce dernier a permis aux différents politiciens et chefs de guerre de s’entendre pour restaurer la paix et démocratiser le pays. La nouvelle Constitution entrée en vigueur le 18 février 2006^4^, a servi de socle à la tenue des élections législatives et présidentielles de 2006, de 2011 et de 2018. Conscient de la nécessité de moderniser l’administration publique et de restructurer ses ressources humaines, le gouvernement de la RDC a initié des réformes institutionnelles visant à amorcer le processus de décentralisation avec plusieurs défis dont celui de l’appropriation^5,6^. La décentralisation a abouti en 2015 au nouveau découpage du territoire national avec un nombre de provinces passant de 11 à 26, dont la ville de Kinshasa, capitale de la RDC, ayant le statut de province. La décentralisation administrative a conféré à la Ville, à la Commune, au Secteur et à la Chefferie la personnalité juridique ainsi que le statut d’Entité Territoriale Décentralisée (ETD).

La décentralisation implique d’une part, le transfert partiel des pouvoirs, des compétences et des responsabilités ainsi que des charges et ressources financières aux Provinces et ETD et d’autre part, la participation des populations dans le processus de prise de décisions. Ainsi, par exemple, les provinces bénéficient de compétences exclusives dans vingt-neuf domaines dont la santé, l’éducation primaire, secondaire et professionnelle, le développement rural et l’agriculture, les affaires coutumières et de compétences concurrentes avec l’État central dans vingt-cinq autres domaines^4^.

Concernant la situation sanitaire, si des progrès sensibles ont été enregistrés pour certains des Objectifs du Millénaire pour le Développement, dans l’ensemble, ceux-ci n’ont cependant pas été totalement atteints^7^ et les indicateurs de la santé et de la nutrition de la RDC figurent parmi les plus bas au monde avec un taux de mortalité infanto-juvénile de 104‰^8^, une mortalité maternelle de 846 décès/100 000 naissances vivantes^8^ et une espérance de vie de 50 ans.

Parallèlement, le niveau intermédiaire du système de santé en RD Congo qui, avant la réforme, intégrait à la fois l’Inspection provinciale de la santé (IPS) et la Division provinciale de la santé (DPS), deux entités pilotées par un seul responsable, le Médecin Inspecteur Provincial, a été mis à l’épreuve. Une étude menée par Kahindo a révélé une évolution temporelle des rôles exercés par le niveau intermédiaire du système sanitaire^9^. En effet, dépourvue de compétences et de financements suffisants, durant les années 80, le niveau intermédiaire s’était progressivement adonné à des fonctions d’inspection et de contrôle, rendant des comptes plus spécifiquement au niveau central du ministère de la Santé et n’exerçant que très faiblement le rôle clé de soutien des zones de santé. A partir de la décennie 90, face à la demande pressante de soutien des équipes des zones de santé, dont l’autonomie de gestion a été mise à rude épreuve suite aux urgences humanitaires consécutives aux guerres, à la nécessité d’intégrer les programmes verticaux et aux logiques de nombreux acteurs, le niveau intermédiaire du système sanitaire a développé des méthodes et des outils pour soutenir la Zone de santé. Ainsi a émergé un modèle subsidiaire du niveau intermédiaire dont l’efficacité perçue était variable selon les provinces^9^, caractérisé par des niveaux variables d’exécution des fonctions managériales dont la coordination, l’encadrement des zones de santé, l’inspection-contrôle, la gestion des ressources, ou la gestion de l’information sanitaire. La nécessité d’adapter les politiques, les normes et les directives sanitaires nationales aux particularités des zones de santé et d’en appuyer l’application, au besoin la contrôler^9^, figure parmi les principales motivations des réformes au niveau intermédiaire. Ces réformes introduites depuis 2015 pour améliorer l’encadrement des Zones de santé consacrent la séparation entre la DPS, une institution décentralisée dépendant du gouvernement provincial, et l’IPS, une institution déconcentrée tributaire du gouvernement central. Elles définissent les rôles que doivent exercer ces structures. Le présent article vise à évaluer l’effet de ces réformes sur la performance du niveau intermédiaire du système de santé dans la province du Sud Kivu.

## Méthode

### Description du terrain d’étude

Située à l’Est de la République Démocratique du Congo, la province du Sud-Kivu compte en 2017 une population estimée à 70 millions d’habitants.

Avant la réforme, le système de santé dans la province comprenait l’IPS, cinq Districts sanitaires, 12 coordinations des programmes spécialisées, 34 Zones de santé, 48 hôpitaux dont un hôpital provincial de référence et 548 centres de santé.

Après la réforme consacrée par l’arrêté ministériel du 03/11/2012, le système de santé provincial est constitué par le ministère provincial de la santé, la division provinciale, l’inspection de la santé, des programmes spécialisés et 34 zones de santé au niveau opérationnel.

### Période et cadre de l’étude

**FIGURE 1:**
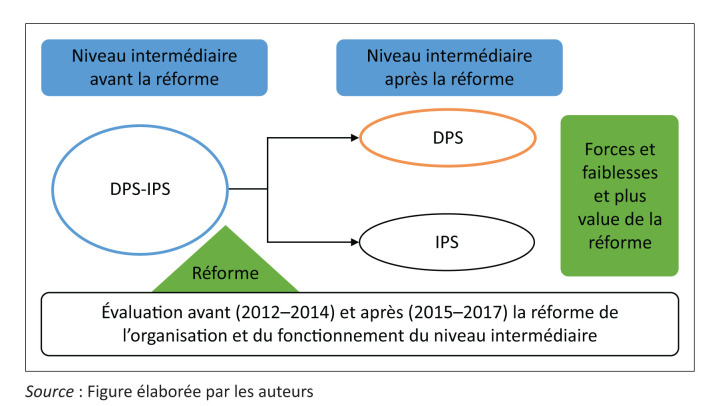
Cadre de l’étude.

L’étude se déroule entre 2012 et 2017, soit trois années avant la décentralisation du secteur de la santé (2012–2014) et trois dernières années après (2015–2017). La Figure 1 synthétise la structuration du niveau intermédiaire avant et après la réforme. Les deux institutions étaient fusionnées avant la réforme et la plupart des fonctions étaient menées par les mêmes acteurs simultanément. Deux institutions distinctes sont issues de la réforme avec des attributions et tâches bien définies.

### Type d’étude et population à l’étude

L’étude est de type évaluatif appliquant des méthodes mixtes. Pour décrire l’organisation et le fonctionnement de l’IPS-DPS avant et après, l’étude a recouru au personnel Cadre de ces institutions afin de collecter des données qualitatives^10^. Les cadres qui ont travaillé durant les trois dernières années dans l’un des bureaux de l’IPS et des programmes spécialisés faisaient partie de la base de l’échantillonnage, de même que de nouveaux cadres, des anciens de la DPS et IPS, des cadres des programmes ainsi que des cadres des ZS encadrées par le niveau intermédiaire au Sud Kivu. Tous les cadres ayant une responsabilité managériale dans l’exercice de la mission du niveau intermédiaire avant et après la réforme ont été systématiquement inclus dans l’étude. Les cadres de la DPS et de l’IPS nouvellement recrutés, ayant moins d’un an d’ancienneté et qui ne faisaient pas partie de l’IPS avant la réforme étaient systématiquement exclus de l’échantillon. Au total, 36 informateurs clés ont été retenus dont 9 cadres de la DPS, 5 cadres de l’IPS, 7 cadres des programmes et 15 cadres des zones (à raison de trois par zone de santé).

### Collecte des données

Trois techniques ont été utilisées pour collecter les données : la revue documentaire, les entrevues individuelles auprès des informateurs clés et l’observation non participante. La triangulation^11^ des informations collectées de ces différentes sources a été appliquée.

La Revue documentaire : elle a consisté à recueillir les informations en rapport avec l’organisation, le fonctionnement et la performance du niveau intermédiaire avant et après la réforme à l’aide d’une fiche préalablement élaborée. Les informations ont été collectées par le chercheur principal à partir des rapports des réunions d’équipe, des plans d’actions opérationnels ; des plans de travail trimestriels, des rapports de supervisions effectués au niveau des différents bureaux de l’institution, des programmes et dans les zones de santé, les rapports des ZS sur l’encadrement du niveau périphérique, des indicateurs de cinq zones choisis aléatoirement sur les 34 ZS de la province (conseils d’administration, autres encadrements dont les zones ont bénéficiées de la part de l’IPS/DPS). Seuls les rapports techniques dans le cadre du fonctionnement, de l’organisation et de l’évaluation des activités avant et après la réforme ont été retenus.

Les entrevues individuelles : elles ont recouru à un questionnaire ouvert ciblant la perception des cadres de la DPS, de l’IPS, des programmes spécialisés et des ZS sur l’organisation, le fonctionnement et l’évaluation des activités du niveau intermédiaire de système de santé avant et après la réforme. Les entrevues ont concerné les 36 informateurs clés décrits plus haut.

L’observation : elle a été non participante, conduite à l’aide d’une grille visant l’appréciation du fonctionnement des institutions issues de la réforme (IPS/DPS). Dans le cadre de cette étude, l’observation a aidé à identifier de l’intérieur de chaque nouvelle entité des faits relatifs à l’organisation et au fonctionnement de l’IPS et de la DPS, notamment le respect des normes et des règlements, la présence des cadres au travail, le temps consacré à l’encadrement des zones de santé au travers de l’analyse des dossiers de ces zones, le mode de prise de décision, la qualité et l’exécution des décisions, ainsi que le niveau de motivation et de satisfaction des cadres. Le chercheur principal a passé trois journées d’observation par institution.

Les données collectées ont concerné la manière dont les fonctions managériales ont été exercées par l’IPS et par la DPS avant et après la décentralisation. Le Tableau 1 présente les données collectées au cours de la période de l’étude (2012–2017)

**TABLEAU 1:** Données quantitatives collectées par fonction managériale et par an.

Fonction managériale	Données collectées	Cible annuelle
Coordination	Nombre de réunions de comité de pilotage du secteur de la santé	2
	Nombre de réunions des groupes thématiques	24
	Nombre de réunions de l’Équipe cadre provinciale (ECP)	48
	Nombre de réunions de conseils d’administration des zones de santé	68
	Nombre de réunions avec les responsables des programmes	4
	Nombre de réunions avec les partenaires techniques et financiers	4
	Nombre de réunions d’appui à l’élaboration des plans d’action annuels des zones de santé	34
Planification	Nombre d’activités réalisées sur nombre d’activités prévues	100%
	Nombre de plans de travail élaborés sur nombre prévu	4
Supervision	Nombre de supervisions des zones de santé	4
	Nombre de supervisions des programmes	4
	Nombre de missions d’encadrement des zones de santé (ZS)	4
Inspection & Contrôle	Nombre de missions d’inspection	4
	Nombre de missions de contrôle	4
	Nombre de missions d’audit	4
Surveillance épidémiologique	Nombre de formations organisées en surveillance épidémiologique intégrée et riposte	34
	Nombre de réunions de surveillance épidémiologique	53
	Nombre de zones de santé avec une promptitude des rapports >80%	34
Information sanitaire	Nombre d’ateliers de formation	68
	Nombre de rapports des ZS reçus Encodage données/zones	34
	Nombre de réunions de validation des données du système national d’information sanitaire (SNIS)	12
	Nombre d’audits de la qualité des données	4
Formation continue	Nombre des personnes formées sur nombre prévu	100%
	Nombre de formations réalisées/nombre prévu	100%
Gestion des ressources et logistique	Respect du manuel de gestion des ressources	100%
	Nombre du personnel en poste sur nombre prévu	100%
	Nombre d’inventaires à jour	2
	Nombre de moyens de ruptures en stock des médicaments dans les ZS	0
	Nombre d’activités financées/Nombre d’activités planifiées	80%
	Nombre de séances d’entretien et de maintenance réalisées/prévues	100%

*Source* : Tableau élaboré par les auteurs

### Analyse des données

L’analyse des données collectées, relatives à l’exercice des fonctions managériales dévolues à l’IPS et à la DPS, a fait recours à deux cadres, à savoir : le cadre normatif du ministère national de la santé publique de la RDC (avant et après la réforme) et le cadre de l’analyse des forces- faiblesses-opportunités et menaces (Analyse FFOM)^12^.

### Cadre normatif avant et après la réforme

Les données collectées ont été comparées au cadre normatif de l’IPS et de la DPS avant et après la réforme. Les écarts observés ont été dégagés et expliqués à l’aide des éléments issus essentiellement des entrevues et de l’observation. Au cours de l’analyse, la situation avant la réforme a été comparée à la situation après la réforme par rapport aux différentes fonctions managériales pour identifier l’existence ou pas de la valeur ajoutée de la réforme. Avant la réforme, 8 fonctions ont été analysées pour l’IPS/DPS à savoir la coordination, la planification, la supervision, l’inspection et le contrôle, la supervision épidémiologique, l’information sanitaire, la formation continue et la gestion des ressources. Après la réforme, l’analyse a porté sur les nouvelles fonctions définies tant pour la DPS que l’IPS. Les 4 fonctions prises en compte pour la DPS sont l’appui et l’encadrement des Zones de santé, la gestion des ressources, l’information sanitaire, la recherche et la communication et l’inspection-contrôle^13^. Ces fonctions regroupent les 8 fonctions d’avant la réforme. Concernant l’IPS, la seule fonction prise en compte après la réforme est l’inspection – contrôle, déclinée en inspection financière et administrative, inspection médicotechnique, inspection pharmaceutique et inspection de l’enseignement des sciences de la santé.

Afin de mieux faire ressortir les valeurs des fonctions managériales du niveau intermédiaire avant et après la réforme durant la période de 2012 à 2017, un score de 10 pour chaque fonction managériale a été introduit. Le score a été attribué par l’équipe des chercheurs en fonction du niveau chiffré d’atteinte de chaque groupe d’indicateurs par fonction managériale, repris dans le Tableau 1. Il s’agit à chaque fois d’une moyenne annuelle des indicateurs spécifiques à la fonction managériale. Le calcul se fait sur la base des informations collectées au niveau de la DPS et de l’IPS. Ainsi, partant des données brutes collectées pendant la revue documentaire des différents rapports des bureaux (à la DPS ) et des rapports annuels de l’IPS des années 2012 à 2017, chaque résultat réalisé a été comparé à la cible annuelle (Tableau 1) pour évaluer le niveau atteint entre 0 et 10. Des scores moyens ont été ensuite calculés par structure (DPS, IPS) avant et après la réforme. La méthode de calcul qui a été appliquée était la même pour le périodes d’avant et d’après la réforme, rendant ainsi fiable ce processus d’évaluation.

### Cadre de l’analyse FFOM

Les forces, les faiblesses, les opportunités et les menaces ont été dégagées à partir des entrevues réalisées sur le fonctionnement de l’IPS et de la DPS avant et après la réforme. Les informateurs clés ont été codifiés en IC1 à IC9 pour la DPS, IC10 à IC14 pour l’IPS, IC15 à IC21 pour les programmes et IC22 à IC36 pour les Zones de santé. Le logiciel QDA Miner Lite a permis de réaliser cette analyse FFOM.

### Limites méthodologiques

La principale limite de cette étude était l’impossibilité d’atteindre l’ensemble des acteurs dans les institutions pour collecter le maximum d’informations. Les autres limites concernent la non-participation des cadres indisponibles et le biais de mémoire pouvant émaner des informateurs clés seniors^14^. Ce biais a été minimisé par l’application de la triangulation des informations. La collecte des données au niveau des Zones de santé n’a pas fait l’objet de la présente étude. Elle aurait permis d’apprécier la tendance de l’évolution des indicateurs sanitaires avant et après la réforme au niveau opérationnel du système de santé et d’évaluer ainsi indirectement le niveau d’encadrement des zones de santé par les structures du niveau intermédiaire. L’évaluation proprement dite de la performance de la DPS et de l’IPS n’a pas non plus fait l’objet de la présente étude ; cette dernière visait à comparer l’évolution des performances avant et après la réforme et non la qualité de la performance. Enfin, la disponibilité, la qualité et la complétude de certains documents remplis en routine et exploités lors de la revue documentaire n’étaient pas maximales.

### Considérations éthiques

Le protocole de recherche avait été validé par le comité de recherche de l’École régionale de santé publique de l’Université Catholique de Bukavu en janvier 2017 ainsi que par l’équipe cadre de la DPS du Sud-Kivu. Un consentement éclairé a été systématiquement obtenu auprès de chaque participant avant chaque entrevue. La confidentialité a été observée tout au long du processus de collecte et d’analyse des données.

### Conflits d’intérêts

Nous n’avons pas de conflit d’intérêt.

## Résultats

### Caractéristiques socio-professionnelles des personnes interrogées

Parmi les 36 personnes interrogées, 42 % travaillent dans les zones de santé où sont principalement déployées les activités de la DPS et de l’IPS ; 92 % sont des hommes ; la moitié sont des cadres (inspecteur, chef de bureau, médecin coordinateur et médecin chef de ZS) et plus du tiers ont une ancienneté dépassant 15 ans (37 %) comme présenté dans le Tableau 2.

**TABLEAU 2:** Tableau des caractéristiques socio-professionnelles des personnes interrogées.

Variables	Effectif (*n* = 36)	Pourcentage
**Institution**		
DPS	9	25,0
IPS	5	13,8
Programmes	7	19,4
Zone de santé	15	41,6
**Sexe**		
Homme	33	91,6
Femme	3	8,3
**Age (années)**		
25–45	18	50,0
45 et plus	18	50,0
**Niveau d’étude**		
Secondaire	0	0,0
Universitaire	19	52,7
Post-univers	17	47,2
**Profession**		
Infirmier	12	33,3
Technicien de développement rural	1	2,7
Administrateur gestionnaire	5	13,9
Pharmacien	2	5,6
Médecin	16	44,4
**Fonction**		
Secrétaire	1	2,8
Nutritionniste	3	8,3
Administrateur gestionnaire	3	8,3
Animateur communautaire	1	2,8
Infirmier superviseur	3	8,3
Pharmacien	2	5,7
Analyste	3	8,3
Assistant programme	3	8,3
Chef de bureau	5	13,9
Inspecteur	4	11,1
Médecin chef de zone	3	8,3
Médecin coordonnateur	5	13,9
**Ancienneté (années)**		
0–5	8	22,2
6–10	8	22,2
11–15	7	19,4
15 et plus	13	36,1

*Source* :

DPS, Division provinciale de la santé ; IPS, l’Inspection provinciale de la santé.

### Scores globaux annuels de réalisation des activités par fonction managériale avant et après la réforme

**FIGURE 2:**
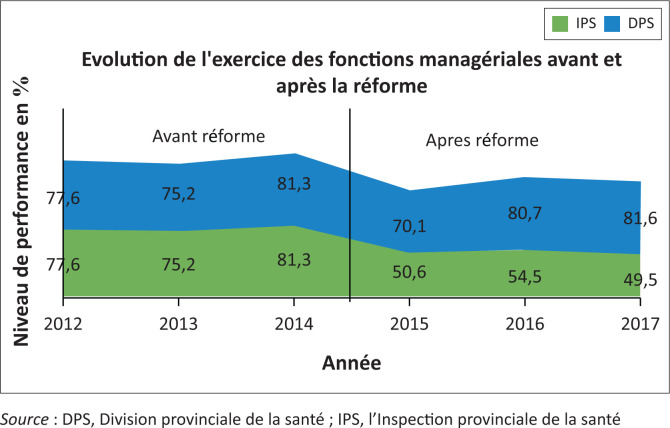
Scores globaux de réalisation des fonctions managériales avant et après la réforme.

Avant la réforme, le niveau d’exécution des activités définissant les fonctions managériales de l’IPS/DPS avait une bonne évolution. Après la réforme, le score de réalisation des activités des fonctions de l’IPS avait sensiblement chuté. La DPS après la réforme avait affiché une bonne évolution des scores de réalisation de ses activités comme illustré par la Figure 2.

#### Perception des personnes interrogées

La bonne évolution de score de réalisation des activités de l’IPS/DPS les trois dernières années avant la réforme est attribuée à la disponibilité des financements, il s’agit de la «belle époque» selon certains cadres interrogés (IC11, IC12, IC13, IC18, IC19). Après la réforme les fonctions managériales de l’IPS ont connu une baisse à cause du faible financement (IC12, IC13), du retard dans la mise en œuvre du processus de réforme et du retard de confirmation de nouveaux cadres de l’IPS à leurs postes (IC11, IC12, IC17, IC18).

A la DPS par contre, les scores moyens de réalisation des fonctions ont mieux évolué avant la réforme (IC18, IC19) grâce au financement des partenaires financiers et techniques et ensuite après la réforme grâce au renforcement du leadership et à la coordination de la DPS par des personnes compétentes désignées sur la base d’un concours (IC1, IC2, IC3, IC4).

«… avant la réforme, c’était la belle époque, des missions, des considérations et du respect de tout le monde du moins les cadres …»IC12

«… au début, la réforme a fait que l’IPS et la DPS soient comme des ennemis. Il faut plutôt considérer qu’ils sont tous dans un même bateau, chacun avec ses fonctions et prérogatives tout en allant vers une destination unique …»IC11

«La réforme a mis de l’ordre dans le travail, les tâches sont bien connues et les profils de postes mais les moyens sont réduits» IC7

### Évolution du niveau de réalisation des activités managériales par la division provinciale de la santé

Le niveau d’exécution des fonctions managériales de la DPS s’est amélioré après la réforme.

#### Perception des personnes interrogées

Les fonctions managériales de la DPS ont évolué de manière satisfaisante après la réforme grâce au renforcement des organes de pilotage (IC1, IC2, IC3, IC4), à la motivation de la nouvelle équipe cadre provinciale recrutée sur la base d’un concours (IC3, IC4, IC5, IC 6, IC7, IC8, IC9) et à la poursuite des financements par des partenaires financiers (tous les informateurs clés). La fonction d’inspection et de contrôle à la DPS crée une certaine confusion avec la même fonction à l’IPS (IC4, IC7, IC12, IC24, IC26, IC27).

«… la DPS a renforcé son leadership par le fonctionnement effectif des organes de pilotage (CPP-SS, secrétariat technique et groupe de travail), les CA des zones, les réunions de CODESA …» IC4

### Évolution du niveau de réalisation des activités managériales par l’Inspection provinciale de la santé

**FIGURE 3:**
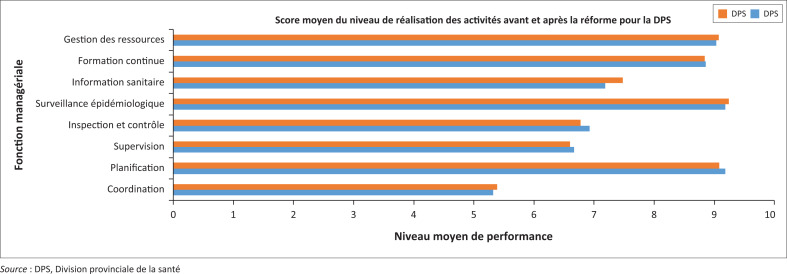
Scores moyens de réalisation des activités des fonctions managériales de la DPS avant et après la réforme.

Comme le montre la figure 3, le score moyen de réalisation des activités de la fonction essentielle d’inspection-contrôle de l’IPS est passé de 6,7/10 avant la réforme à 5,8/10 après la réforme. Cette seule fonction essentielle retenue après la réforme a régressé de 13 %, qu’il s’agisse de l’inspection financière et administrative, de l’inspection médicotechnique, de l’inspection pharmaceutique ou de l’inspection de l’enseignement des sciences de la santé.

#### Perception des personnes interrogées

La régression du score d’exécution de la fonction de l’inspection-contrôle de l’IPS après la réforme est associée à un très faible apport financier pour soutenir les activités de l’IPS et à une démotivation des cadres en place dont la confirmation à leurs nouveaux postes a pris beaucoup de temps (C12, IC13, IC15, IC17, IC18). Avant la réforme, la structure DPS/IPS comptait la présence de cadres expérimentés (IC2, IC4, IC8) et un financement important (IC6, IC8, IC18) entre 2012 et 2014. La fonction d’inspection et de contrôle, activité majeure avant la réforme au niveau des zones de santé, n’était plus assurée efficacement par l’IPS après la réforme, faute de financement(IC2, IC11, IC12).

«la séparation entre IPS et DPS a été bénéfique, car les tâches d’un chacun sont bien connues, mais le financement pose problème surtout pour l’IPS et le respect des textes …» IC3

### Forces et valeurs ajoutées de la réforme

Les cadres interrogés attestent que les organes de coordination ont été renforcés avec la mise en place d’un comité provincial de pilotage du secteur de la santé (CPP-SS) et des groupes de travail thématiques. Ces structures constituent une valeur ajoutée de la réforme car elles permettent de rassembler tous les experts de la province avec l’autorité politique provinciale principale (IC1, IC2, IC3, IC4, IC5, IC6, IC7, IC8, IC9, IC11) afin de débattre des problèmes sanitaires prioritaires et d’évaluer les interventions sectorielles. La réforme a également offert la possibilité d’un découpage interne des zones de santé afin de résoudre le problème d’accessibilité géographique en favorisant la construction des centres de santé plus proches de la population dans les nouvelles ZS (IC4, IC12, IC13)

Deux forces sont reconnues à la réforme, (1) l’introduction d’un livre d’emploi à l’IPS et à la DPS avec une description claire des tâches et attributions de chaque agent (IC4, IC5, IC6, IC7, IC11, IC12) et (2) l’alignement des partenaires financiers de la province à un contrat unique de financement des activités de la DPS en vue de l’exécution du plan annuel de travail (IC4, IC5, IC9, IC18), une sorte de fonds commun pour soutenir les interventions sanitaires dans la province.

«Le rapprochement vers les zones, les tâches et attributions bien définies dans la DPS et IPS, tout l’argent des partenaires passent par la DPS : c’est le contrat unique qui posera des soucis au retrait des partenaires». IC18

«La réforme actuelle est déjà une bonne chose et un acquit, mais à celà, il faut aussi ajouter le service autonome de paie à instaurer comme c’est le cas au Ministère de l’Enseignement primaire et secondaire …»IC11

### Faiblesses de la réforme

Selon les personnes interrogées, le financement de la réforme administrative au niveau intermédiaire du système de santé était faible (IC1, IC2, IC6, IC8). Par conséquent, la mise en œuvre de la dite réforme était difficile (IC1, IC7, IC8). Par ailleurs, les difficultés d’adaptation éprouvées par les cadres suite à la réforme (IC5, IC6, IC7), étaient à la base de la démotivation de ces derniers (IC1, IC2, IC3, IC4). En plus, cette démotivation était aggravée par le fait suivant : quelques agents n’étaient pas immédiatement reconnus suite à la réforme. Ils étaient ainsi privés de salaire de l’État et de la prime du Fonds-Mondial, un partenaire technique et financier important au niveau intermédiaire (IC1, IC2, IC3, IC4, IC5, IC6, IC7, IC8). Enfin, la réforme n’a pas apporté de solution à l’absence d’une centrale de distribution régionale des médicaments dans la province du Sud-Kivu, laissant ainsi courir sur les marchés locaux non régulés de faux médicaments (IC1, IC2, IC3, IC4, IC5, IC11, IC12).

«… la charrue a précédé le bœuf : la réforme devait d’abord disposer des ressources matérielles, financières pour son accompagnement …» IC6

«… la réforme est mieux faite dans son ensemble, ce qui fait un peu défaut, ce sont les moyens financiers promis qui ne sont pas mobilisés du côté de l’État …» IC8

### Opportunités de la réforme pour le renforcement du système de santé

La réforme a permis la mise en place d’un contrat unique pour le financement des activités. Ce contrat unique est assorti d’un contrat de performance au sein de la DPS(IC3,IC4,IC6,IC7). Cette innovation, consistant à introduire une nouvelle approche de gestion des ressources financières, favorise l’émulation des cadres. Elle permet également d’apprécier à juste titre les efforts de chaque cadre ou de chaque groupe de cadres selon la fonction managériale spécifique et les objectifs institutionnels à atteindre (IC1, IC5, IC6, IC7).

«Les personnels sont maintenant à la hauteur de leurs tâches et le contrat de performance existe qui indique une amélioration des prestations dans toutes les institutions du niveau intermédiaire» IC7

### Menaces de la réforme

La fuite des cerveaux, c’est-à-dire, les départs des cadres bien formés, a été mentionnée. Ces départs ont été occasionnés par l’insatisfaction des attentes suscitées par la réforme, la perception d’une carrière brisée suite à la réforme et le mauvais climat de travail (déconsidérations, rémunération insignifiante, conflits interpersonnels, interférences politiques dans les nominations) (IC2, IC3, IC4, IC6, IC7, IC11, IC12, IC13). La faible application des textes et normes règlementaires constitue également une menace (IC18, IC19).

«… cette réforme s’est faite sans l’avis des bénéficiaire …, a créé la misère des certains agents retrouvés sans poste … a entraîné le départ d’autres agents plus qualifiés et devenus mécontents …» IC23

«Par rapport aux deux institutions issues de la réforme la cohabitation reste un défi majeur mais les efforts sont fait de part et d’autre pour améliorer le climat de travail. Mais le grand problème, c’est au niveau périphérique (ZS, HGR, AS) où la réforme n’est pas encore bien comprise …» IC18

«… Au Sud-Kivu il n’y a pas vraiment de collaboration entre la DPS et l’IPS, … C’est à l’IPS de jouer ce rôle de faire respecter les textes et les normes règlementaires, car souvent il y a de jeux de Ping Pong …» IC19

Aussi, le serveur du logiciel DHSI2 se trouvant en dehors du pays est perçu comme une menace non résolue par la réforme du fait d’une absence totale de contrôle sur la manipulation extérieure des données, parfois sensibles, collectées par la DPS (IC3, IC6, IC7).

Malgré la création de l’organe de pilotage de l’action sanitaire provinciale (CPP-SS), des interférences politiques dans la prise de certaines décisions d’affectation des ressources humaines ont persisté, mettant en déroute les procédures de recrutement, notamment au niveau des Zones de santé (IC1, IC2, IC4, IC7, IC11)

«Oui, la réforme a été positive mais je déplore l’interférence politique dans les nominations de certains cadres …» IC2

## Discussion

La présente étude avait pour objectif d’évaluer l’effet de la réforme sur la performance des structures intermédiaires du système de santé au Sud-Kivu. D’une part, la période évaluée était courte (moins de 5 ans) pour apprécier les résultats, contrairement à d’autres études d’évaluation qui considèrent plusieurs années^15^. D’autre part, il était difficile de retrouver certains informateurs clés partis de la province. Nous discutons dans les paragraphes qui suivent les caractéristiques socio-professionnelles des cadres interrogés, le niveau de performance réalisé avant et après la réforme par l’IPS et la DPS. Nous discutons également, au travers l’exécution des fonctions managériales, les forces, des faiblesses, des menaces et des opportunités de la réforme telles qu’identifiées au cours de l’étude.

### Caractéristiques socio-professionnelles

Au cours de cette étude, nous avons trouvé que parmi les 36 personnes interrogées, 42 % travaillent dans les zones de santé où sont principalement déployées les activités de la DPS et de l’IPS ; 92 % sont des hommes et 8 % des femmes. Cette faible représentation des femmes dans ces structures sanitaires s’expliquerait par le fait que la situation sociale de la plupart des cadres femmes est défavorable aux fonctions administratives ; bon nombre de femmes n’ayant pas atteint un niveau académique de maîtrise pour mériter des postes des cadres. Nos résultats corroborent ceux d’autres études^16,17^. La moitié de personnes interrogées sont des cadres (inspecteur, chef de bureau, médecin coordinateur et médecin chef de ZS) et plus du tiers ont une ancienneté dépassant 15 ans (37 %). Ceci est lié d’une part à l’accumulation de l’expériences au fil du temps dans le système et d’autre part à la nature des tâches du niveau intermédiaire d’un système de santé nécessitant essentiellement des cadres.

### Niveau de performance réalisé par l’IPS et la DPS avant et après la réforme

La performance d’exécution des fonctions managériales a une évolution en dents de scie au cours des trois dernières années avant la réforme. Après la réforme, la DPS améliore sa performance, avec le soutien financier des partenaires financiers et la mise en place d’une nouvelle équipe cadre provinciale^18^ alors que l’IPS régresse. Cette régression semble associée à la non priorisation de l’IPS en tant que système lors de la réforme, contrairement à la DPS ; n’ayant pas bénéficié d’emblée d’un financement conséquent pour son fonctionnement^19,20^. Toutefois, il y a lieu d’espérer que le processus de la réforme déjà initiée aboutira et que la fonction « inspection – contrôle » de l’IPS sera développée de manière efficace. Le processus de réorganisation du système de soins ne peut se faire que dans la durée. Ce type de changement s’accompagne de négociations, d’appropriation et d’un processus d’apprentissage. Il faudra alors une approche longitudinale pour apprécier les effets de cette réforme dans la durée^21^.

La réforme a renforcé la coordination de l’action sanitaire par la mise en place de nouveaux arrangements institutionnels^22,23^, notamment la création de deux organes (CPP-SS, Groupes thématiques) où participent les deux institutions pour les rendre plus efficaces, efficients et équitables conformément aux objectifs de la décentralisation du système de santé^18,24^.

La planification des activités au cours du processus de la réforme a été un succès. Néanmoins, le faible taux de réalisation des activités enregistré est dû à la faible mobilisation des ressources, surtout financières. Ces ressources financières proviennent principalement des partenaires techniques et financiers. Ainsi la réforme étant un processus national et régalien, il revient au gouvernement d’assumer l’augmentation du budget du secteur de la santé, notamment à 15 %, tel que recommandé dans la déclaration des chefs d’État à l’issue du Sommet d’Abuja sur le financement de la santé^25,26^. La réforme exige un personnel compétent, expérimenté, formé continuellement pour faire face à de nouvelles méthodes ou à de nouveaux protocoles introduits dans la prise en charge des soins pour la performance du système de santé. Malheureusement, la fuite des cerveaux suite à la recherche de conditions meilleures constitue un facteur défavorable au développement du système de santé des pays en voie de développement^27^ et notamment dans le cas de l’IPS et de la DPS.

Le maintien du métier « inspection-contrôle » au sein de la DPS sème la confusion avec celui de l’IPS. En effet, le bureau d’Inspection contrôle de la DPS contrôle l’application effective des politiques nationales, des directives, des stratégies et des normes dans les zones de santé, les établissements classés, les officines pharmaceutiques et les établissements d’enseignements des sciences de santé, occasionnant ainsi une confusion avec la fonction d’« inspection-contrôle » exercée de l’IPS. En effet, au niveau de l’IPS, la fonction inspection-contrôle a les mêmes attributions. Au travers de cette fonction, la DPS est appelée à assurer un audit interne tandis que l’IPS assure l’audit externe. Ces notions devront être davantage clarifiées dans les directives nationales et les politiques locales.

### Forces, faiblesses, menaces et opportunités de la réforme

L’alignement des partenaires financiers de la province à un contrat unique de financement des activités de la DPS, soutenu par un plan annuel de travail, offre l’opportunité d’améliorer la performance de l’Équipe cadre provinciale^28^. Le faible financement de l’État dans le secteur de la santé demeure la principale faiblesse de la réforme que ce dernier a initié, l’exposant à des manœuvres réduites face aux problèmes de santé^27^, à un faible contrôle du système de santé et à la perte de son pouvoir de régulation, dont une des conséquences est la circulation des médicaments de mauvaise qualité.

Les cadres recrutés, qui avaient espéré une carrière fructueuse au sein de la DPS et de l’IPS, réformées à la suite des conditions de travail incitatives ont vite fait face à des perspectives d’emploi peu optimistes. Les conditions de travail, lorsqu’elles sont défavorables, génèrent la démotivation, la fuite des cerveaux, voire la contre-performance des systèmes sanitaires^29^. La menace de la poursuite de la fuite des cerveaux à la DPS et à l’IPS demeure. Il faudra envisager d’améliorer les conditions de travail, de renforcer la productivité et la performance des cadres disponibles, d’améliorer la rétention du personnel et d’impliquer les pays du Nord ainsi que les organisations internationales qui recrutent les cadres des pays en développement^29,30^ dans le développement des ressources humaines de qualité, de plus en plus rares.

Enfin, des études ultérieures sont indiquées, pour évaluer la qualité des interactions de l’équipe de la DPS avec les équipes cadres des zones de santé. Une étude menée dans la ville de Lubumbashi en RDC^31^ a montré que, quand ces interactions ne sont pas optimales, leurs effets sur les performances des zones de santé sont limités.

## Conclusion

Considérant les deux périodes de l’étude (avant et après la réforme), la performance de la DPS a été améliorée tandis que celle de l’IPS a régressé, sous l’angle de l’exécution des activités qui définissent les fonctions managériales du niveau intermédiaire du système de santé en RDC. Le faible financement, notamment celui de l’État, a constitué un goulot d’étranglement pouvant compromettre la réussite de la réforme. Cependant, cette dernière a offert l’opportunité d’améliorer la performance de la DPS grâce à la mise en place d’un contrat unique de financement des partenaires assorti d’un plan annuel de travail. Elle a aussi permis le renouvellement de l’équipe cadre provinciale sur la base d’un mécanisme de recrutement par concours.

## Leçons apprises

1. Le succès d’une réforme dans le secteur de la santé est tributaire d’une préparation préalable avec les parties prenantes et d’un financement adéquat pour sa mise en œuvre.
2. La mise en œuvre de la réforme doit être coordonnée, accompagnée et supervisée par un organe où siègent des acteurs clés de la réforme.
3. La réforme constitue une des opportunités pour améliorer la performance des systèmes de santé.
4. Dans les pays où le fonctionnement des systèmes de santé dépend fortement de l’aide internationale, la réforme permettrait de centraliser l’aide pour financer de manière transparente les plans de développement ou d’action des systèmes de santé concernés.
